# Diagnostik und Therapie der Otitis externa necroticans

**DOI:** 10.1007/s00106-025-01690-5

**Published:** 2025-12-12

**Authors:** Verena Strasser, Lea Stecher, Gerlig Widann, Teresa Steinbichler

**Affiliations:** 1 Universitätsklinik für Hals‑, Nasen- und Ohrenheilkunde, Medizinische Universität Innsbruck, Anichstr. 35, 6020 Innsbruck, Österreich; 2 Universitätsklinik für Hals‑, Nasen- und Ohrenheilkunde, Kopf- und Halschirurgie Innsbruck, Medizinische Universität Innsbruck, Anichstr. 35, 6020 Innsbruck, Österreich

**Keywords:** Osteomyelitis, Diabetes mellitus, Hirnnervenerkrankungen, Positronenemissionstomographie, *Pseudomonas aeruginosa*, Osteomyelitis, Diabetes mellitus, Cranial nerve diseases, Positron-emission tomography, *Pseudomonas aeruginosa*

## Abstract

Die Otitis externa necroticans ist eine seltene, potenziell lebensbedrohliche, invasive Infektion des äußeren Gehörgangs. Sie tritt vorwiegend bei älteren, immunsupprimierten Patient:innen – v. a. mit Diabetes mellitus – auf. Die Erkrankung kann in eine Osteomyelitis der Schädelbasis übergehen, ggf. mit schwerwiegenden Komplikationen wie Hirnnervenparesen, Meningitiden oder Sinusvenenthrombosen. Häufigster Erreger ist* Pseudomonas aeruginosa*, zunehmend auch invasive Pilzinfektionen. Die therapeutische Basis bildet eine gezielte systemische antimikrobielle Behandlung über mehrere Wochen. In komplizierten Verläufen kann eine chirurgische Intervention erforderlich sein. Zur Verlaufsbeurteilung und Therapiekontrolle eignet sich die Positronenemissionstomographie (^18^F‑Fluordesoxyglukose-PET) in Kombination mit Felsenbein-Computertomographie oder -Magnetresonanztomographie. Lebenslange, engmaschige HNO-fachärztliche Nachsorge ist essenziell, um Rezidive frühzeitig zu erkennen.

## Abstract (en)

Necrotizing otitis externa (NOE) is a rare, potentially life-threatening invasive infection of the external auditory canal that predominantly affects elderly and immunocompromised individuals—particularly those with diabetes mellitus. The disease may progress to skull base osteomyelitis and can lead to severe complications such as cranial nerve palsy, meningitis, or dural sinus thrombosis. The most common causative pathogen is *Pseudomonas aeruginosa*, although invasive fungal infections are increasingly recognized. The primary therapeutic strategy involves extended administration of pathogen-specific systemic antimicrobial agents. Surgical intervention may be required for complicated clinical courses. For monitoring disease progression and evaluating the therapeutic response, fluorodeoxyglucose positron-emission tomography (18F-FDG-PET) in combination with computed tomography (CT) or magnetic resonance imaging (MRI) of the temporal bone has proven valuable. Lifelong closeknit otolaryngological follow-up is essential to detect recurrences early.

## Lernziele

Nach der Lektüre dieses Beitrags …

- sind die in der Lage, eine Otitis externa necroticans zuverlässig zu erkennen,
- können Sie Risikopatient:innen rechtzeitig identifizieren,
- wissen Sie die richtigen diagnostischen Schritte einzuleiten,
- können Sie die korrekten therapeutischen Maßnahmen veranlassen.

## Kasuistik

Ein 80-jähriger Patient stellte sich mit einer neu aufgetretenen **peripheren Fazialisparese**

Periphere Fazialisparese

links sowie seit einer Woche bestehenden, intensiven, linksseitigen Kopfschmerzen und **putrider Otorrhö**

Putride Otorrhö

**Abb. 1:**
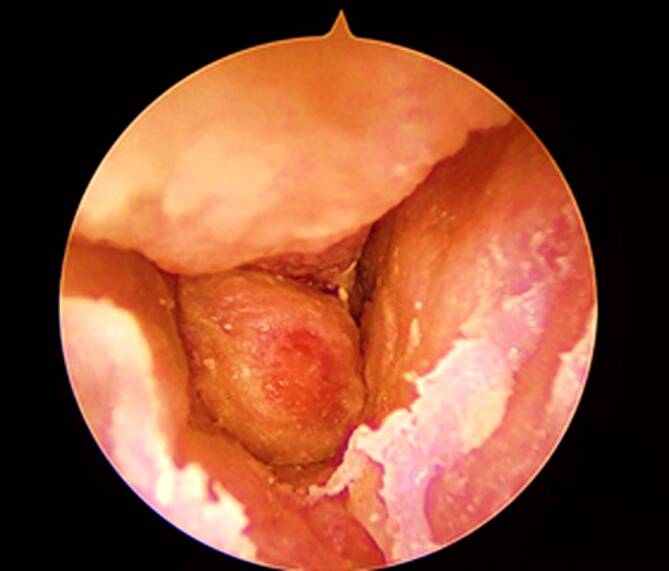
Typischer ohrmikroskopischer Befund einer Otitis externa necroticans (OEN) mit diffuser Entzündung des Gehörgangs und Granulationsgewebe im hinteren Bereich. Trommelfell vollständig von der Granulation überdeckt, daher nicht beurteilbar

**Abb. 2:**
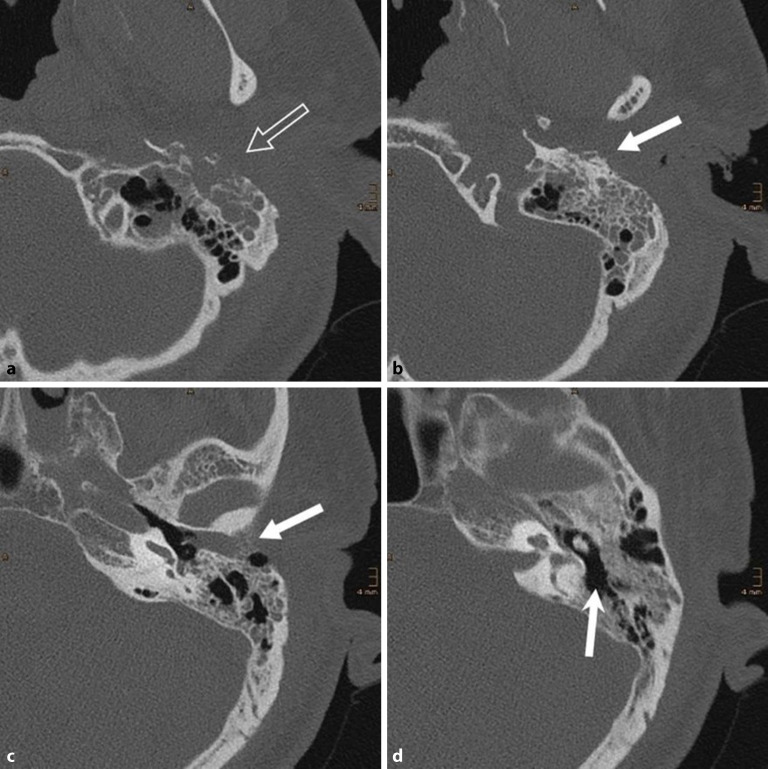
Felsenbein-Computertomographie (CT) bei Otitis externa necroticans bei einem 80-jährigen Mann mit **a** nekrotischer Zerstörung des vorderen Mastoids (*offener, nicht ausgefüllter Pfeil*), **b,c** entzündlicher Verlegung des äußeren Gehörgangs und nekrotischer Arrosion des unteren Randes (*weißer Pfeil*) sowie **d** luftgefülltem Paukenraum und Antrum (*weißer Pfeil mit offener Spitze*)

Otorrhö vor. Otoskopisch zeigte sich ein geröteter Gehörgang mit nahezu vollständig verlegendem Granulationsgewebe vor dem Trommelfell (Abb. 1). Vor dem Hintergrund eines bekannten Typ-2-Diabetes mellitus mit inadäquater Stoffwechseleinstellung (HbA1c 8,1 %) ergab sich der klinische Verdacht auf eine Otitis externa necroticans (OEN). Ein mikrobiologischer Abstrich wurde entnommen, außerdem erfolgte eine hochauflösende Computertomographie (CT) des Felsenbeins. Diese zeigte nekrotische Arrosionen des Gehörgangsbodens und des vorderen Mastoids (Abb. 2). Aufgrund der bereits eingetretenen Hirnnervenbeteiligung wurde eine **Mastoidektomie**

Mastoidektomie

durchgeführt sowie eine systemische **antibiotische Therapie**

Antibiotische Therapie

mit Ceftazidim begonnen. Zusätzlich erfolgte eine topische Behandlung mit ciprofloxacinhaltigen Ohrentropfen und eine intensivierte diabetologische Mitbetreuung. Im mikrobiologischen Abstrich wurde ein auf die eingeleitete Therapie sensibler *Pseudomonas aeruginosa *nachgewiesen. Nach 14-tägiger i.v.-Therapie wurde der Patient in gebessertem Zustand mit oraler Fortführung (Levofloxacin) entlassen. Jedoch kam es 4 Wochen nach Entlassung zu einem Rezidiv mit erneuter Zunahme der Otorrhö und Schmerzsymptomatik. Eine weitere 2‑wöchige i.v.-Behandlung wurde erforderlich, gefolgt von einer 8‑wöchigen oralen Erhaltungstherapie. Der Patient blieb anschließend über 2 Jahre unter engmaschiger HNO-ärztlicher Kontrolle rezidivfrei.

## Verlauf der Otitis externa necroticans

Die Otitis externa necroticans (OEN) ist eine potenziell **lebensbedrohliche Erkrankung**

Lebensbedrohliche Erkrankung,

die durch eine invasive Infektion des äußeren Gehörgangs gekennzeichnet ist und zu einer **destruierenden Perichondritis**

Destruierende Perichondritis

und **Osteomyelitis**

Osteomyelitis

der lateralen Schädelbasis führen kann. Aufgrund ihrer hohen Mortalität wird sie häufig auch als „maligne“ Otitis externa bezeichnet. Komplikationen umfassen Hirnnervenparesen, intrakranielle Abszesse, Meningitiden sowie Sinusvenenthrombosen. Die Erkrankung tritt vorwiegend bei **immunsupprimierten Patient:innen**

Immunsupprimierte Patient:innen

über 65 Jahren auf, insbesondere mit schlecht kontrolliertem Diabetes mellitus. *Pseudomonas aeruginosa* ist der am häufigsten isolierte Erreger [1, 2, 3].

**Abb. 3:**
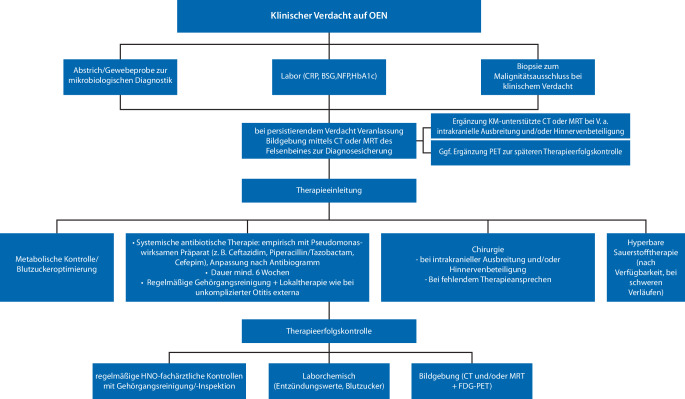
Schematische Darstellung der Diagnostik und Therapie der Otitis externa necroticans. *BSG *Blutsenkungsgeschwindigkeit, *CRP *C‑reaktives Protein,* CT* Computertomographie, *FDG* ^18^F‑Fluordesoxyglukose, *KM* Kontrastmittel, *MRT* Magnetresonanztomographie, *NFP* Nierenfunktionsparameter, *OEN* Otitis externa necroticans, *PET *Positronenemissionstomographie

Der vorliegende Artikel bietet eine kompakte Übersicht über diese seltene, aber häufig mit komplizierten Verläufen assoziierte Form der Gehörgangsentzündung mit Fokus auf Diagnostik und Therapie zur Erleichterung des klinischen Managements. Die Abb. 3 zeigt zusammenfassend eine schematische Darstellung der wichtigsten diagnostischen und therapeutischen Schritte.

### Merke

Die Otitis externa necroticans ist selten, geht aber häufig mit kompliziertem Verlauf einher.

## Definition

In der Literatur wird wiederholt auf die uneinheitliche Datenlage und die Notwendigkeit der Etablierung standardisierter diagnostischer Kriterien hingewiesen [1]. Zum aktuellen Zeitpunkt stehen keine evidenzbasierten Leitlinien zur OEN zur Verfügung. Die Tab. 1 zeigt einen nach Hodgson et al. modifizierten Vorschlag für Diagnosekriterien einer OEN [4].

**Tab. 1:** Diagnosekriterien einer Otitis externa necroticans. (Mod. nach Hodgson et al. [5])

OEN definitiv vorliegend bei Erfüllung *aller *4 folgenden Kriterien	
1.	Otalgie und Otorrhö *oder *Otalgie und vorhergehend Otorrhö in der Anamnese
2.	Granulationsgewebe *oder* diffuse Entzündung des Gehörgangs
3.	Histopathologischer Ausschluss von Malignität bei klinischem Verdacht
4.	Radiologische Befunde passend zur OEN in der CT und/oder MRT (knöcherne Arrosionen/Knochenmarködem im Felsenbein und Weichteilentzündung des äußeren Gehörgangs)

*CT* Computertomographie, *MRT* Magnetresonanztomographie, *OEN* Otitis externa necroticans

Unter den Autoren besteht weitgehend Konsens darüber, dass zur vollständigen Diagnostik **bildgebende Verfahren**

Bildgebende Verfahren

in Form einer Computertomographie (CT) oder Magnetresonanztomographie (MRT) des Felsenbeins vorliegen müssen. Für die seltene Konstellation, dass die Bildgebung keine wegweisenden Befunde zeigt, schlagen Hodgson et al. folgende, unter Tab. 2 präsentierte Diagnosekriterien vor [4].

**Tab. 2:** Diagnosekriterien einer Otitis externa necroticans bei fehlenden bildgebenden Zeichen. (Mod. nach Hodgson et al. [5])

OEN möglicherweise vorliegend bei einer schweren Gehörgangsentzündung ohne bildmorphologische Zeichen einer OEN in der CT oder MRT, wenn *alle *3 der folgenden Kriterien erfüllt sind	
1.	Otalgie und Otorrhö *oder *Otalgie und vorhergehend Otorrhö in der Anamnese
2.	Granulationsgewebe *oder* diffuse Entzündung des Gehörgangs
3.	Eines der folgenden Kriterien:
	Immundefizienz (insbesondere Diabetes mellitus)
	Nachtschmerz
	Erhöhte laborchemische Entzündungsparameter (Blutsenkung, CRP) ohne erkennbare andere Ursache
	Therapieversagen auf topische Antiinfektiva und Ohrtoilette > 2 Wochen

*CRP *C‑reaktives Protein,* CT* Computertomographie, *MRT* Magnetresonanztomographie, *OEN* Otitis externa necroticans

## Anatomie und Pathogenese

Der Gehörgang besteht aus einem äußeren knorpeligen und einem inneren knöchernen Anteil. Der knorpelige, rinnenförmige Anteil wird am Gehörgangsboden von mehreren kleinen Spalten, den **Santorini-Spalten**

Santorini-Spalten

durchzogen. Über diese kann die Entzündung bei invasiven Infektionen des äußeren Gehörgangs auf umliegendes Gewebe, z. B. die Glandula parotis und das Kiefergelenk, übergreifen. Eine weitere Ausbreitungspforte kann ein persistierendes Foramen tympanicum, auch **Huschke-Foramen**

Huschke-Foramen

genannt, darstellen. Hierbei handelt es sich um einen anatomische Varietät, bei der es durch einen unvollständigen Verschluss des knöchernen Gehörgangs in der embryologischen Entwicklung zu einer persistierenden Lücke am Gehörgangsboden in Richtung Fossa infratemporalis kommt [6].

### Merke

Die Ausbreitung der Entzündung bei der OEN erfolgt typischerweise über Knorpelspalten am Gehörgangsboden.

## Komplikationen

Komplikationen der OEN drohen, wenn sich die Entzündung auf das umliegende Weich- und Knochengewebe ausbreitet. Sie gleichen denen der akuten Mastoiditis und können potenziell lebensbedrohlich sein.

Insbesondere bei einer Osteomyelitis der lateralen Schädelbasis kann es frühzeitig zu **Hirnnervenausfällen**

Hirnnervenausfälle

kommen. Am häufigsten ist der N. facialis (VII) betroffen. Bei schwereren Verläufen können auch die kaudalen Hirnnerven wie der N. glossopharyngeus (IX), N. vagus (X), N. accessorius (XI) und N. hypoglossus (XII) beteiligt sein. Bei einer weiteren Ausbreitung der Entzündung in den intrakraniellen Raum besteht das Risiko für die Entwicklung einer **septischen Sinusvenenthrombose**

Septische Sinusvenenthrombose,

intrakranielle Abszessbildungen, subdurale und epidurale Empyeme sowie das Auftreten einer Meningitis [7].

Breitet sich die Entzündung vom Mastoid nach kaudal in die Halsweichteile aus, kann es zu einer phlegmonösen Infiltration des retro- oder parapharyngealen Raums kommen, mit potenzieller Folge einer **Mediastinitis**

Mediastinitis

oder einer septischen Thrombophlebitis der V. jugularis interna. Bei Durchbruch der Entzündung an der Mastoidspitze in den M. sternocleidomastoideus entsteht der „**Bezold-Abszess**

Bezold-Abszess

“. Weitere mögliche Komplikationen umfassen Sepsis, **Labyrinthitis**

Labyrinthitis

mit konsekutivem Hörverlust und Gleichgewichtsstörungen, Duraperforationen mit Liquorfistelbildung sowie bleibende neurologische Defizite infolge verzögerter Diagnostik und Therapie [5, 8].

Die häufigsten Komplikationen der OEN sind:

- Schädelbasisosteomyelitis mit Hirnnervenparesen (N. VII, N. IX, N. X, N. XI, N. XII)
- Septische Sinusvenenthrombose
- Intrakranielle Abszesse/Empyeme
- Meningitis
- Phlegmone/Abszesse der Halsweichteile mit retro-/parapharyngealer Ausbreitung

### Merke

Zu den häufigsten Komplikationen der OEN gehören Schädelbasisosteomyelitis, Hirnnervenparesen, septische Sinusvenenthrombose, intrakranielle Abszesse.

## Epidemiologie

Im Gegensatz zur unkomplizierten Otitis externa, welche zu den häufigsten Vorstellungsgründen in der HNO-Heilkunde zählt, handelt es sich bei der OEN um ein seltenes Krankheitsbild. Aufgrund der spärlichen Datenlage und mangels einheitlich angewandter Diagnosekriterien kann keine genaue Angabe zur Inzidenz gegeben werden. **Prädisponierende Grunderkrankungen**

Prädisponierende Grunderkrankungen

wie Diabetes mellitus und chronische Niereninsuffizienz begünstigen die Entstehung einer OEN. Angesichts der demografischen Entwicklung und der weltweit steigenden Prävalenz des Diabetes mellitus ist künftig auch mit einer Zunahme der OEN zu rechnen. Der Erkrankungsgipfel liegt typischerweise im **höheren Lebensalter**

Höheres Lebensalter,

insbesondere bei Personen über 65 Jahren, wobei Männer häufiger betroffen sind als Frauen [3, 8]. Auch bei Kindern kann eine OEN auftreten, insbesondere bei bestehendem Diabetes mellitus. Wegen der im kindlichen Schädel näheren Lage des N. facialis zu den Santorini-Spalten kommt es hier häufiger und früher zu Fazialisparesen als beim Erwachsenen [9].

### Merke

Hauptrisikofaktoren für die Entwicklung einer OEN sind männliches Geschlecht, Alter > 65 Jahre, Immunsuppression, insbesondere Diabetes mellitus.

## Diagnostik

### Klinische Untersuchung

Patient:innen mit OEN präsentieren sich typischerweise mit starken Ohren- und **Kopfschmerzen**

Kopfschmerzen

und Otorrhö, welche auf die topische Therapie nicht suffizient ansprechen. Häufig wird über eine **nächtliche Schmerzzunahme**

Nächtliche Schmerzzunahme

berichtet, und durch eine entzündliche Beteiligung des Kiefergelenks können verstärkt Schmerzen beim Kauen auftreten. Der typische ohrmikroskopische Befund zeigt zusätzlich zu einer diffusen Entzündung des Gehörgangs auch Granulationsgewebe (Abb. 1; [10]).

### Differenzialdiagnosen

Die seltene Differenzialdiagnose des **Gehörgangskarzinoms**

**Abb. 4:**
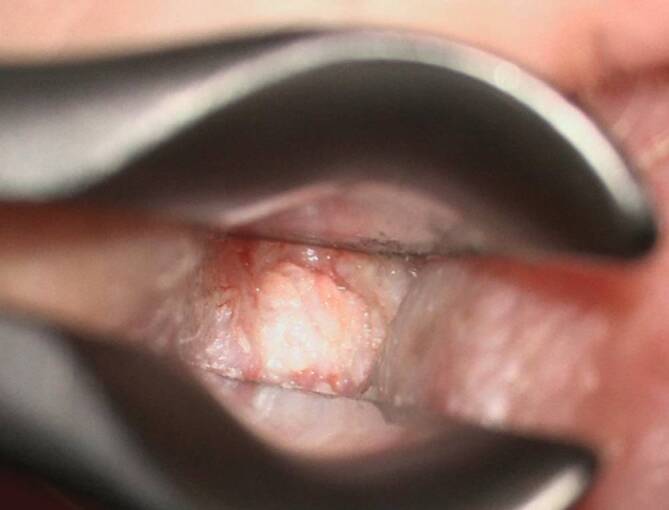
Bild eines Plattenepithelkarzinoms des äußeren Gehörgangs. Aufgrund der klinischen Ähnlichkeit zur Otitis externa necroticans (OEN) bei unklarer Befundkonstellation frühzeitige Gewebeprobe zur histopathologischen Abklärung empfohlen

Gehörgangskarzinom

sollte bei jedem Fall einer OEN mitbedacht werden und bei potenziellem Verdacht eine Biopsie zur histopathologischen Aufarbeitung entnommen werden (Abb. 4; [11]). Eine weitere Differenzialdiagnose stellt das **Gehörgangscholesteatom**

**Abb. 5:**
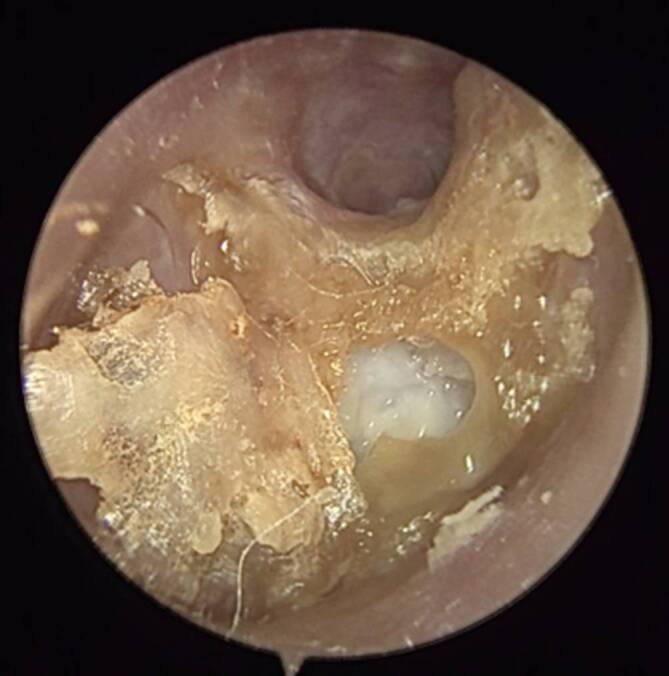
Bild eines Gehörgangscholesteatoms mit Stufenbildung am Gehörgangsboden, freiliegendem Knochen (*weißlich*) und aufgelagerten Keratinkrusten (*gelb*)

Gehörgangscholesteatom

dar (Abb. 5). Hier kommt es typischerweise zu einer von festen Krusten bedeckten Aushöhlung im Bereich des Gehörgangsbodens mit freiliegendem Knochen. Eine sichere Abgrenzung zur OEN erfordert bildgebende Verfahren, anhand derer eine Ausbreitung der Entzündung über die Grenzen des Gehörgangs hinaus ausgeschlossen werden kann.

#### Merke

Das Gehörgangskarzinom stellt die wichtigste Differenzialdiagnose der OEN dar und muss im Verdachtsfall rasch mittels Biopsie ausgeschlossen werden.

### Laborchemische Untersuchung

Beim klinischen Verdacht auf eine OEN sollte eine Blutabnahme zur Bestimmung des C‑reaktiven Proteins (CRP), der Blutsenkungsgeschwindigkeit (BSG), der Nierenfunktionsparameter und des glykierten Hämoglobins (HbA1c) erfolgen. Takata et al. beschreiben anhand einer systematischen Literaturauswertung, dass bei etwa 2 Dritteln der Patient:innen eine Erhöhung der BSG und bei etwas mehr als der Hälfte eine Erhöhung des CRP zum Zeitpunkt der Erstdiagnose festgestellt wurde. Somit kommt diesen **Entzündungsparametern**

Entzündungsparametern

im Rahmen der initialen Diagnosestellung nur eine untergeordnete Bedeutung zu, sie können aber im weiteren Verlauf der Behandlung Hinweise auf das Therapieansprechen geben [1].

### Mikrobiologie

Bei jeder therapierefraktären Otitis externa sollte frühestmöglich ein mikrobiologischer **Abstrich**

Abstrich

zur Keimbestimmung gewonnen werden. Zusätzlich sollten **Gewebeproben**

Gewebeproben

von etwaiger Granulation zur mikrobiologischen Untersuchung entnommen werden, da oberflächliche Abstriche, v. a. nach bereits erfolgtem topischem Therapieversuch mit Antiseptika, häufig nicht alle Erreger erfassen [12].

Das Keimspektrum wird in der überwiegenden Mehrzahl der Fälle durch gramnegative Erreger, insbesondere *Pseudomonas aeruginosa*, dominiert. **Pseudomonaden**

Pseudomonaden

zeichnen sich durch eine hohe Affinität zu feuchtem Milieu sowie eine ausgeprägte Fähigkeit zur Biofilmbildung und Gewebeinvasion aus. Seltener können auch andere **gramnegative Bakterien**

Gramnegative Bakterien

wie *Proteus spp., Klebsiella spp.* oder *Escherichia coli* beteiligt sein. Insbesondere bei stark immunsupprimierten Patient:innen spielen auch zunehmend invasive **Pilzinfektionen**

Pilzinfektionen

eine entscheidende Rolle, hier werden überwiegend die Gattungen Candida und Aspergillus nachgewiesen [13].

#### Merke

***Pseudomonas aeruginosa*** ist der am häufigsten isolierte Keim bei einer OEN.

### Bildgebung

Zur **Diagnosesicherung**

Diagnosesicherung

einer OEN eignen sich die CT und MRT des Felsenbeins gleichermaßen. Zur exakten Beurteilung des **Ausdehnungsmusters**

Ausdehnungsmuster

kann eine Kombination der beiden Verfahren sinnvoll sein, wobei im klinischen Alltag die zumeist schneller und kostengünstiger verfügbare CT bevorzugt eingesetzt wird. Bei Vorliegen von Zeichen einer fortgeschrittenen Ausbreitung der Entzündung sollten jedenfalls zusätzlich eine kontrastmittelunterstützte CT oder MRT zur Beurteilung der intrakraniellen Ausbreitung ergänzt werden [14].

Zur Kontrolle des **Therapieerfolgs**

Therapieerfolg

sind CT und MRT nur bedingt geeignet, da sie auch nach Abklingen der Infektion häufig noch reaktive Gewebeveränderungen zeigen. **Nuklearmedizinische Untersuchungsverfahren**

Nuklearmedizinische Untersuchungsverfahren

können in diesem Zusammenhang einen entscheidenden Mehrwert bieten, da sie Rückschlüsse auf die aktuelle Krankheitsaktivität ermöglichen. Andere Parameter wie klinische Symptome und Laborwerte (z. B. CRP oder BSG) sind in ihrer Aussagekraft begrenzt und korrelieren nicht zuverlässig mit dem Infektionsstatus. Während früher v. a. Knochen- und Galliumszintigraphien eingesetzt wurden, hat sich mittlerweile die Positronenemissionstomographie mit Fluordesoxyglukose (^18^F‑FDG-PET) als Methode der Wahl zur Beurteilung des Therapieerfolgs etabliert. Sie dient zunehmend auch als Entscheidungsgrundlage für den optimalen Zeitpunkt zur Beendigung der antibiotischen Behandlung [15].

#### Merke

CT und MRT zeigen bei einer OEN typische Befunde. Zur Beurteilung der Krankheitsaktivität eignet sich eine funktionelle Bildgebung mittels FDG-PET.

## Therapie

Die Therapie der OEN basiert auf einer gezielten systemischen und lokalen antimikrobiellen Behandlung und der adäquaten Kontrolle prädisponierender Grunderkrankungen. In therapierefraktären Fällen und bei Hirnnervenbeteiligung kann zusätzlich eine chirurgische Intervention indiziert sein. Der Stellenwert der hyperbaren Sauerstofftherapie als adjuvante Therapieoption bei schweren Verläufen wird in der Literatur diskutiert, wobei hierfür aufgrund der niedrigen Evidenz aktuell keine allgemeingültige Empfehlung besteht.

### Antimikrobielle Therapie

Für die empirische systemische Therapie vor Kenntnis der auslösenden Keime wird eine **Pseudomonas-wirksame Therapie**

Pseudomonas-wirksame Therapie

empfohlen. Diese sollte unter Berücksichtigung der bestehenden Komorbiditäten, Kontraindikationen und lokalen Resistenzsituation gewählt werden, nach Möglichkeit in Absprache mit einer Fachabteilung für Infektiologie.

Lodhi et al. beschreiben in einer systematischen Literaturauswertung, dass an den meisten Zentren initial eine i.v.-Kombinationstherapie eingeleitet und im weiteren Verlauf auf eine orale Monotherapie mit einem Fluorchinolon umgestellt wird. Die bisher häufig initial eingeleitete empirische Kombinationstherapie aus Ceftazidim und Ciprofloxacin ist vor dem Hintergrund aktueller Sicherheitsbewertungen und Resistenzentwicklungen kritisch zu betrachten. Nach dem 2019 erschienenen Rote-Hand-Brief des Bundesinstituts für Arzneimittel und Medizinprodukte (BfArM) unterliegen Fluorchinolone, insbesondere Ciprofloxacin, aufgrund des Risikos potenziell irreversibler, schwerwiegender Nebenwirkungen (u. a. Tendopathien, Neuropathien, Aortenaneurysmen und -dissektionen) einer strengen Indikationsstellung und dürfen nur bei gesicherter bakterieller Ursache und fehlenden Therapiealternativen eingesetzt werden [16]. Die aktuelle S2k-Leitlinie zur Antibiotikatherapie bei HNO-Infektionen empfiehlt daher ausdrücklich, eine systemische Fluorchinolontherapie nicht empirisch ohne gesicherten Erregernachweis und nicht bei unkomplizierten Verläufen oder fehlender Pseudomonas-Resistenzlage einzusetzen. Für die empirische Initialtherapie bei Verdacht auf OEN wird gemäß aktueller Evidenzlage bevorzugt eine **i.v.-Monotherapie**

i.v.-Monotherapie

mit einem **Anti-Pseudomonas-β-Laktam-Antibiotikum**

Anti-Pseudomonas-β-Laktam-Antibiotikum

(z. B. Ceftazidim, Piperacillin/Tazobactam oder Cefepim) empfohlen. Eine Kombinationstherapie mit Ciprofloxacin sollte ausschließlich bei nachgewiesenem Fluorchinolon-sensiblem *Pseudomonas aeruginosa*, fehlenden Kontraindikationen und nach individueller Risiko-Nutzen-Abwägung unter Berücksichtigung der genannten Sicherheitswarnungen erfolgen. Nach Vorliegen des Antibiogramms ist die Therapie strikt deeskalierend anzupassen [17].

#### Merke

Als empirische Therapie sollte ein Pseudomonas-wirksames Präparat gewählt und bei vorliegender Keimbestimmung dem Antibiogramm angepasst werden.

Hinsichtlich der Behandlungsdauer lässt die derzeitige Datenlage keine allgemeingültige Empfehlung zu. In der Regel wird jedoch eine **Mindesttherapiedauer von 6 Wochen**

Mindesttherapiedauer von 6 Wochen

angeraten. In schweren und komplizierten Fällen kann eine Verlängerung der antibiotischen Behandlung über mehrere Monate erforderlich sein [18].

Bei einer fungalen nekrotisierenden Otitis externa gilt **Voriconazol**

Voriconazol

als Mittel der Wahl. Aufgrund zunehmender Resistenzraten von Aspergillus- und Candida-Spezies kann jedoch häufig eine Umstellung der Therapie oder eine Kombination mit weiteren Antimykotika erforderlich werden. Zudem treten nicht selten Mischinfektionen von bakteriellen und fungalen Erregern auf, was eine individuell angepasste, interdisziplinär abgestimmte Therapie notwendig macht [13].

Die topische Therapie bei OEN wird in der Literatur kaum behandelt. Obligat sind **regelmäßige Gehörgangsreinigungen**

Regelmäßige Gehörgangsreinigungen.

Unterstützend können zur Hemmung der lokalen Entzündungsreaktion und zur Schmerzlinderung getränkte Gazestreifen zur Anwendung kommen, wobei keine Vergleichsstudien zwischen antibiotischer und rein antiseptischer Lokaltherapie (z. B. Alkohol) vorliegen [19].

#### Merke

Fluorchinolone dürfen bei der OEN nur bei schweren Verläufen, mikrobiologischem Erregernachweis und fehlenden Therapiealternativen eingesetzt werden.

### Chirurgie

Grundsätzlich gelten sowohl ein **fehlendes Therapieansprechen**

Fehlendes Therapieansprechen

auf konservative Maßnahmen sowie die Beteiligung von Hirnnerven als Indikation zur chirurgischen Intervention. Das Ausmaß der gewählten chirurgischen Maßnahme sollte sich an der klinischen Symptomatik und den in der Bildgebung von der Entzündung und Nekrosen betroffenen Arealen richten. Mögliche chirurgische Maßnahmen reichen von **lokalem Débridement**

Lokales Débridement

über Mastoidektomien (Canal-Wall-up oder Canal-Wall-down) bis hin zu Fazialisdekompressionen und **Petrosektomien**

Petrosektomien

[20].

### Hyperbare Sauerstofftherapie

Die hyperbare Sauerstofftherapie (HBST) beruht auf physikalischen Gasgesetzen und ermöglicht durch erhöhten Umgebungsdruck eine gesteigerte Aufnahme sowie Verteilung von Sauerstoff im Gewebe. Darüber hinaus konnten antimikrobielle und **wundheilungsfördernde Effekte**

Wundheilungsfördernde Effekte

nachgewiesen werden, u. a. durch die Stimulation von Phagozyten, Fibroblasten und angiogenetischen Faktoren. Für eine Reihe von Indikationen – wie Kohlenmonoxidvergiftung, Osteoradionekrose, Dekompressionskrankheit und schwere Verbrennungen – existieren klare Leitlinienempfehlungen zur Anwendung der HBOT. Für die Therapie der Otitis externa necroticans (OEN) wurde bislang aufgrund unzureichender Evidenz keine Empfehlung durch die Fachgesellschaften ausgesprochen [21]. Einzelne Studien berichten jedoch über positive Effekte, sodass die HBST angesichts des geringen Risiko- und Nebenwirkungsprofils als adjuvante Therapieoption bei schweren Verläufen in Erwägung gezogen werden kann [22, 23].

## Nachsorge

Eine **engmaschige HNO-fachärztliche Nachsorge**

Engmaschige HNO-fachärztliche Nachsorge

ist bei der OEN entscheidend, um Rezidive frühzeitig zu erkennen und rechtzeitig zu behandeln. Bei Diabetikern sollten zusätzlich auf eine **konsequente Blutzuckerregulierung**

Konsequente Blutzuckerregulierung

geachtet werden.

## Fazit für die Praxis

- Die Otitis externa necroticans (OEN) ist eine potenziell lebensbedrohliche Infektion des Gehörgangs, welche zu einer Osteomyelitis der lateralen Schädelbasis führen kann.
- Meist tritt die OEN bei älteren, immunsupprimierten Patient:innen auf.
- Eine frühe Diagnosestellung ist entscheidend, um Komplikationen (Hirnnervenparesen, intrakranielle Abszesse, Meningitiden, Sinusvenenthrombosen) zu verhindern.
- Eine empirische antibiotische Therapie sollte mit einer Pseudomonas-wirksamen Kombination (z. B. Ceftazidim + Ciprofloxacin) eingeleitet und frühestmöglich antibiogrammgerecht angepasst werden.
- Klinische Symptome und Laborwerte sind zur Beurteilung der Krankheitsaktivität unzuverlässig, die ^18^F‑Fluordesoxyglukose-Positronenemissionstomographie-Computertomographie (^18^F‑FDG-PET-CT) ermöglicht eine verlässliche Beurteilung des Therapieerfolgs und unterstützt die Entscheidung zum Behandlungsende.
- Die antibiotische Therapie muss über mindestens 6 Wochen, in schwereren Fällen häufig auch über mehrere Monate fortgeführt werden, um Rezidive zu verhindern.
- Chirurgische Eingriffe sind bei Hirnnervenbeteiligung und intrakranieller Ausbreitung zusätzlich indiziert.
- Betroffene Patient:innen sollten lebenslang an engmaschigen HNO-fachärztlichen Nachsorgekontrollen teilnehmen, um Rezidive frühzeitig zu erkennen.

## CME-Fragebogen

Welcher der folgenden Keime wird am häufigsten bei einer Otitis externa necroticans isoliert?

*Pseudomonas aeruginosa*

*Candida albicans*

*E. coli*

*Staphylococcus aureus*

*Moraxella catarrhalis*

An welchem Hirnnerv kommt es am häufigsten bei einer Otitis externa necroticans (OEN) zu einer Funktionseinschränkung?

N. abducens

N. facialis

N. trigeminus

N. vagus

N. vestibulocochlearis

Ein 80-jähriger Patient mit insulinpflichtigem Diabetes mellitus wird vom niedergelassenen HNO-Facharzt mit seit 2 Wochen auf antiseptische Lokaltherapie refraktärer Otitis externa an die HNO-Ambulanz überwiesen. Im extern entnommen Abstrich wurde *Pseudomonas aeruginosa* nachgewiesen. Welchen der folgenden Schritte leiten Sie als Nächstes ein?

Fazialis-Elektromyographie (EMG)

Chirurgisches Débridement des Gehörgangs

Computertomographie (CT) des Felsenbeins

Vestibulometrie

Sonographie der Halsweichteile

Welcher der folgenden Computertomographie(CT)-Befunde spricht am ehesten für das Vorliegen einer Otitis externa necroticans (OEN)?

Weichgewebsvermehrung im Prussak-Raum

Abgerundetes Skutum

Knöcherne Arrosion des Gehörgangsbodens

Ossifikation der Cochlea

Osteolyse des Processus condylaris mandibulae

Welche der folgenden anatomischen Strukturen spielt eine zentrale Rolle in der Ausbreitung der Entzündung bei der Otitis externa necroticans (OEN)?

Santorini-Spalten

Foramen jugulare

Pars flaccida

Jacobson-Anastomose

Stenon-Gang

Welche Patientengruppe ist typischerweise von einer Otitis externa necroticans (OEN) betroffen?

Kinder mit Trisomie 21

Ältere, immunsupprimierte Patient:innen

Jugendliche mit Mittelohrentzündung

Schwangere Frauen

Leistungssportler

Welche Bildgebung eignet sich am besten zur Verlaufsbeurteilung bei der Otitis externa necroticans (OEN)?

Computertomographie (CT) des Felsenbeins

Magnetresonanztomographie (MRT) des Felsenbeins

Konventionelle Röntgenaufnahme

^18^F‑Fluordesoxyglukose-Positronenemissionstomographie (^18^F‑FDG-PET)

Sonographie

Welche Therapieoption kann bei therapierefraktärem Verlauf zusätzlich erwogen werden?

Low-Level-Laser-Therapie

Vitamin-D-Supplementierung

Extrakorporale Stoßwellentherapie

Plasmapherese

Hyperbare Sauerstofftherapie

Ein 60-jähriger Patient stellt sich mit seit 3 Wochen persistierenden starken Ohrenschmerzen in der HNO-Praxis vor. Es bestehen keine Komorbiditäten, insbesondere kein Diabetes mellitus. Der Gehörgang zeigte sich subtotal stenosiert und mit einer weißlichen Masse mit unruhiger Oberfläche ausgefüllt. Auf Kontakt mit dem Sauger kommt es direkt zu einer leichten Blutung. Ein mikrobiologischer Abstrich zeigt sich steril. Welchen Schritt veranlassen Sie als Nächstes?

Einleitung einer Lokaltherapie mit Fluorochinolon-haltigen Ohrentropfen.

Systemische antibiotische Therapie mit Amoxicillin/Clavulansäure.

Veranlassung einer ^18^F‑Fluordesoxyglukose-Positronenemissionstomographie (^18^F‑FDG-PET).

Biopsie zum Malignitätsausschluss.

Chirurgisches Débridement.

Welche der folgenden Erkrankungen/Symptome zählt zu den typischen Komplikationen einer Otitis externa necroticans (OEN)?

Myokarditis

Guillain-Barré-Syndrom

Neuropathia vestibularis

Tinnitus

Septische Sinusvenenthrombose
